# Kaiso is highly expressed in TNBC tissues of women of African ancestry compared to Caucasian women

**DOI:** 10.1007/s10552-017-0955-2

**Published:** 2017-09-08

**Authors:** Blessing I. Bassey-Archibong, Shawn M. Hercules, Lyndsay G. A. Rayner, Desiree H. A. Skeete, Suzanne P. Smith Connell, Ian Brain, Adetola Daramola, Adekunbiola A. F. Banjo, Jung S. Byun, Kevin Gardner, Jonathan Dushoff, Juliet M. Daniel

**Affiliations:** 1 Department of Biology, McMaster University, Hamilton, ON Canada; 2 Department of Pathology, Queen Elizabeth Hospital (QEH), Bridgetown, Barbados; 3 Faculty of Medical Sciences, The University of the West Indies, Cave Hill Campus, Bridgetown, Barbados; 4 Department of Radiation Oncology, Queen Elizabeth Hospital (QEH), Bridgetown, Barbados; 5 Department of Pathology and Molecular Medicine, McMaster University, Hamilton, ON Canada; 6 Department of Anatomic and Molecular Pathology, Lagos University Teaching Hospital (LUTH), Lagos, Nigeria; 7 Genetics Branch, National Institute of Health, Bethesda, MD USA

**Keywords:** Kaiso, TNBC, Women of African ancestry, Breast cancer racial disparity

## Abstract

**Purpose:**

Triple-negative breast cancer (TNBC) is most prevalent in young women of African ancestry (WAA) compared to women of other ethnicities. Recent studies found a correlation between high expression of the transcription factor Kaiso, TNBC aggressiveness, and ethnicity. However, little is known about Kaiso expression and localization patterns in TNBC tissues of WAA. Herein, we analyze Kaiso expression patterns in TNBC tissues of African (Nigerian), Caribbean (Barbados), African American (AA), and Caucasian American (CA) women.

**Methods:**

Formalin-fixed and paraffin embedded (FFPE) TNBC tissue blocks from Nigeria and Barbados were utilized to construct a Nigerian/Barbadian tissue microarray (NB-TMA). This NB-TMA and a commercially available TMA comprising AA and CA TNBC tissues (AA-CA-YTMA) were subjected to immunohistochemistry to assess Kaiso expression and subcellular localization patterns, and correlate Kaiso expression with TNBC clinical features.

**Results:**

Nigerian and Barbadian women in our study were diagnosed with TNBC at a younger age than AA and CA women. Nuclear and cytoplasmic Kaiso expression was observed in all tissues analyzed. Analysis of Kaiso expression in the NB-TMA and AA-CA-YTMA revealed that nuclear Kaiso H scores were significantly higher in Nigerian, Barbadian, and AA women compared with CA women. However, there was no statistically significant difference in nuclear Kaiso expression between Nigerian versus Barbadian women, or Barbadian versus AA women.

**Conclusions:**

High levels of nuclear Kaiso expression were detected in patients with a higher degree of African heritage compared to their Caucasian counterparts, suggesting a role for Kaiso in TNBC racial disparity.

**Electronic supplementary material:**

The online version of this article (doi:10.1007/s10552-017-0955-2) contains supplementary material, which is available to authorized users.

## Introduction

Breast cancer (BCa) is a complex disease that occurs mostly in females and is a leading cause of female deaths worldwide [1–3]. The triple-negative breast cancer (TNBC) subtype accounts for a disproportionate number of BCa deaths due to its highly aggressive nature and metastatic tendencies [4–6]. As the name implies, triple-negative tumors represent a subset of breast tumors that are negative for the estrogen receptor (ER), progesterone receptor (PR), and human epidermal growth factor receptor-2 (HER2) [7]. Most TNBC are classified as basal-like cancers and are generally characterized by high histologic/nuclear grade, increased rate of recurrence, and a greater frequency of epidermal growth factor receptor (EGFR) amplification, p53 mutations, and breast cancer type 1 (BRCA1) mutations [7, 8]. Due to their triple-negative status for ER, PR, and HER2, TNBCs lack targeted-treatment options, and cannot be treated with hormonal (Tamoxifen) or anti-HER2 therapies [7].

There is increasing evidence that TNBC occurs more frequently in young premenopausal African and AA women compared to Caucasian women [7, 9–14]. For example, Stark and colleagues reported that among Ghanaian BCa cases, there was a TNBC prevalence of ~82% compared to the USA where TNBC prevalence was ~33% and ~10% among AA and CA cases, respectively [11]. Similarly, Agboola et al. reported a high incidence of TNBC among BCa cases in Nigerian women (~48%) compared with British women (~14%) [14]. The trend of high TNBC prevalence in AA and African females strongly suggests an ancestral genetic predisposition to TNBC in women of African ancestry (WAA) [15–17]. More disturbing, however, is the poor survival rate of AA TNBC patients compared with Caucasian TNBC patients [10, 18], which underscores the urgency to identify potential prognostic or diagnostic TNBC biomarkers in WAA.

Recent studies have found a correlation between increased nuclear expression of the transcription factor Kaiso and poor overall survival of AA breast cancer and prostate cancer patients compared to their Caucasian counterparts [19, 20]. These data hint at a role for Kaiso in the racial disparity in outcomes associated with breast and prostate cancer. Kaiso was first identified as a binding partner of the E-cadherin catenin cofactor—p120-catenin [21]. Kaiso is a dual-specificity transcription factor and member of the POZ-ZF family of transcription factors [21–25] that are implicated in vertebrate development and tumorigenesis. Kaiso has been most often characterized as a transcriptional repressor [26], but some studies indicate that Kaiso can also function as a transcriptional activator [27, 28]. Notably, several Kaiso target genes identified to date (*cyclinD1, matrilysin, E*-*cadherin*) have been linked to tumor onset, invasion, and metastasis [29–31].

Since its discovery, Kaiso has been implicated in the poor prognostic outcomes of several cancers including colorectal, non-small cell lung cancer, prostate, pancreatic ductal adenocarcinoma, and TNBC [20, 32–35]. Studies from our lab and others indicates that Kaiso plays both pro-oncogenic and tumor suppressive roles in several human cancers [19, 20, 33, 34, 36–38]. Notably, in addition to being implicated in racial disparities in breast cancer outcomes, high Kaiso expression correlates significantly with ER-α negativity, and the aggressiveness of basal/TNBCs [35, 38]. To date however, no studies have specifically examined and compared Kaiso expression and subcellular localization in TNBC tissues from WAA, who have the highest prevalence and worst outcomes from TNBC compared to Caucasian women. In this retrospective study, we evaluated Kaiso expression in TNBC specimens from Nigerian, Barbadian, AA, and CA patients. We found that nuclear Kaiso expression was significantly increased in TNBC tissues of Nigerian, Barbadian, and AA patients compared with their Caucasian counterparts. While there was no significant difference in nuclear Kaiso expression in TNBC tissues of Nigerian versus Barbadian patients (who have a higher percentage of African ancestry compared to AA), we found significantly more nuclear Kaiso expression in Nigerian versus AA patients, and a trend towards higher nuclear Kaiso expression in Barbadian versus AA patients. Collectively, these findings suggest that Kaiso may play a role in the racial disparity associated with TNBC in WAA.

## Methods

### Study population and characteristics of tumor samples

FFPE TNBC tissue blocks of 28 Nigerian TNBC patients diagnosed between 2011 and 2013 at the Lagos University Teaching Hospital (LUTH), Nigeria, and 46 Barbadian TNBC patients diagnosed between 2002 and 2011 at the Queen Elizabeth Hospital (QEH), Barbados were obtained from the archives of the Department of Anatomic and Molecular Pathology at LUTH and the Department of Pathology at QEH after approval by LUTH and QEH Ethics committees, respectively. The FFPE specimens were then shipped to the Developmental Histology Lab at the Yale Pathological Tissue Services (YPTS), Yale University (Connecticut, New Haven, USA), where they were hematoxylin and eosin (H&E) stained for histopathological confirmation, before tumor areas from each FFPE tissue block were selected for the construction of a Nigerian and Barbadian TNBC tissue microarray (NB-TMA). ER, PR, and HER2 status of the Nigerian tissues were confirmed by immunohistochemistry (IHC) conducted at LUTH, while ER, PR, and HER2 status of the Barbadian tissues were confirmed by IHC conducted at QEH, Barbados, the Human Tissue Resource Center (Chicago, IL, USA) or the Immunohistochemistry Lab at the University of Miami, Miller School of Medicine (Clinical Research Building, Miami, FL, USA). Any sample with less than 1% staining for ER and PR was scored negative; likewise, 0 or +1 for HER2 was considered negative. Available clinico-pathological data (age, tumor pathology, lymph node involvement, and grade) were retrieved from the hardcopy pathology reports at LUTH and QEH, and are summarized in Table 1.

**Table 1:** Clinico-pathological characteristics and analysis of study participants

	Nigerian (%) *n* = 28	Barbadian (%) *n* = 46	African American (%) *n* = 20	Caucasian American (%) *n* = 43	*χ* ^2^ value	*p* value
Age (years)						
≤50	20 (71.4%)	21 (45.7%)	6 (30.0%)	10 (23.3%)	16.89	0.0007
>50	5 (17.9%)	25 (54.3%)	7 (35.0%)	27 (62.8%)		
Unknown^a^	3 (10.7%)	0 (0.0%)	7 (35.0%)	6 (13.9%)		
Grade						
1	5 (17.9%)	0 (0.0%)	2 (10.0%)	13 (30.2%)	63.59	<0.0001
2	8 (28.6%)	9 (19.6%)	11 (55.0%)	21 (48.9%)		
3	10 (35.7%)	35 (76.1%)	0 (0.0%)	1 (2.3%)		
Unknown^a^	5 (17.8%)	2 (4.3%)	7 (35.0%)	8 (18.6%)		
Stage T						
T1–T2	6 (21.4%)	18 (39.1%)	7 (35.0%)	23 (53.5%)	30.52	<0.0001
T3–T4	11 (39.3%)	1 (2.2%)	1 (5.0%)	0 (0.0%)		
Unknown^a^	11 (39.3%)	27 (58.7%)	12 (60.0%)	20 (46.5%)		
Stage N						
N0	4 (14.3%)	11 (23.9%)	7 (35.0%)	26 (60.5%)	10.23	0.02
N1-N3	11 (39.3%)	10 (21.7%)	13 (65.0%)	12 (27.9%)		
Unknown^a^	13 (46.4%)	25 (54.4%)	0 (0.0%)	5 (11.6%)		

^a^ Unknown cases were exempted from analysis

For the AA and CA patient population, we utilized the Yale tissue microarray 347 (YTMA-347), which was generated at the Yale Developmental Histology Lab, and comprised of 20 AA and 43 CA usable TNBC specimens that were diagnosed at the Yale-New Haven Hospital, Connecticut, USA between 1996 and 2004. ER, PR, and HER2 status were determined by IHC at the Yale Developmental Histology Lab. The clinico-pathological features of the YTMA-347 cohort are summarized in Table 1.

### Immunohistochemistry

5-μm tissue sections prepared from the NB-TMA tissue block and the purchased YTMA-347 tissue slides were de-paraffinized by warming at 60 °C for 20 min, followed by immersion in xylenes for 10 min. Tissue sections were then rehydrated in descending ethanol dilutions before they were subjected to heat antigen retrieval in a low pH buffer (pH 6.0) solution (DAKO, Glostrup, Denmark). Endogenous biotin, biotin receptors, and avidin binding sites on tissues were subsequently blocked using the Avidin/Biotin blocking kit (Vector Laboratories, Inc., Burlingame, CA, USA), while endogenous peroxidase activity was quenched by treatment with 3% hydrogen peroxide. Tissue slides were stained with mouse anti-Kaiso 6F monoclonal (1:10,000; [39]) or mouse anti-human cytokeratin clones AE1/AE3 monoclonal (1:500; Dako North America, Inc., Carpinteria, CA, USA) primary antibodies overnight at 4  °C, followed by secondary antibody incubations at room temperature for 2 h with biotinylated donkey anti-mouse secondary antibody (Vector Labs; 1:1000). Tissues were subsequently incubated in Vectastain (Vector Labs) for 30 min, rinsed in 1X PBS, and then incubated in diaminobenzidine (DAB) (Vector Labs) for 10 min. Counterstaining was achieved by incubating tissues in Harris hematoxylin (Sigma) for 10–60 s, followed by rinsing in tap water or as described in [40]. Slides were then dehydrated in ascending alcohol dilutions, and cleared with two rounds of xylenes before being mounted using Polymount (Polysciences Inc., Warrington, PA, USA). Negative control staining data were achieved by slide incubation with secondary antibodies only. Images of stained slides were captured using the Aperio Slide scanner (Leica Biosystems, ON, Canada). Stained tissues were scored blindly by two Pathologists, and the scores averaged to give a final score value. The intensity of staining was scored as 0, 1, 2, or 3 representing no, mild, moderate, or high staining intensity. The modified histochemical score (H-score) system was then used to generate the total score for each tissue with values spanning 0–300 using the formula: 3 × (percentage of cells with high intensity staining (3+) + 2 × (percentage of cells with moderate intensity staining (2+) + 1 × (percentage of cells with mild intensity staining (1+) for each slide.

### Statistical analysis

GraphPad Prism statistical software (GraphPad Software Inc., La Jolla, CA, USA) was used for all statistical analyses. Standard unpaired Student’s *t* test with Welch’s correction was used for pairwise comparison of means. Chi square analysis was used to assess the difference in clinico-pathological features between the Nigerian, Barbadian, AA, and CA cohorts. Data are presented as mean ± SEM where applicable. For all statistical tests, *p* values <0.05 denote statistical significance.

## Results

### Clinico-pathological characteristics of study participants

**Fig. 1:**
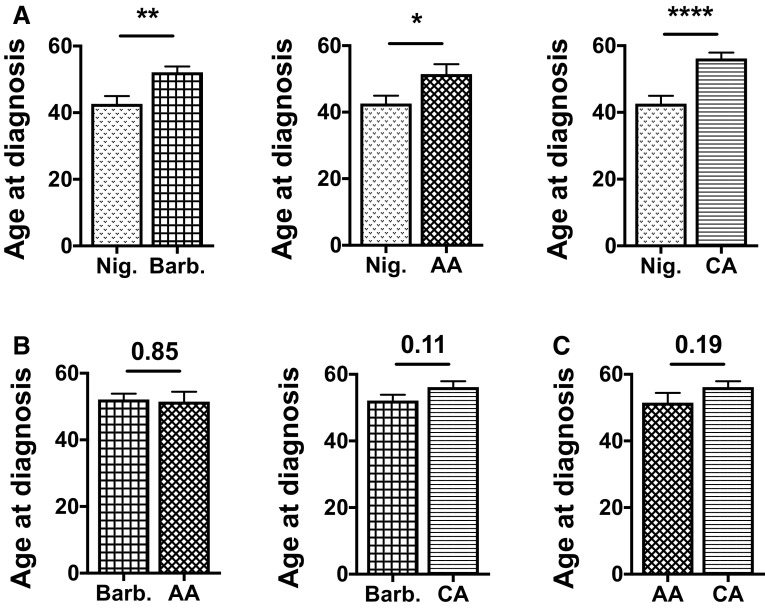
Nigerian women are diagnosed with TNBC at younger ages than Barbadian, AA, and CA women. **a** The mean age at diagnosis for Nigerian TNBC patients was 42.6 years (*n* = 25) compared with 52.1 years for Barbadian women (*n* = 46), 51.5 years for AA women (*n* = 13), and 56.2 years for CA women (*n* = 37). No significant differences were observed between the mean age at diagnosis for Barbadian versus AA and CA TNBC patients (**b**) and for AA versus CA patients (**c**). ^*^ *p* < 0.05, ^**^ *p* < 0.005, ^****^ *p* < 0.0001

This retrospective study involved a total of 28 Nigerian, 46 Barbadian, 20 African American (AA), and 43 Caucasian American (CA) TNBC patients. The mean age at time of diagnosis for Nigerian women was 42.6 years compared to 52.1 years for Barbadian women (*p* = 0.002), 51.5 years for AA women (*p* = 0.03), and 56.2 years for CA women (*p* < 0.0001; Fig. 1a). Comparison of the mean age at diagnosis between Barbadian, AA, and CA patients yielded no statistical significance (Fig. 1b, c). The percentage of younger women who presented with TNBC at time of diagnosis was significantly higher for the Nigerian cohort (71.4%; *n* = 20) compared with the Barbadian (45.7%; *n* = 21), AA (30.0%; *n* = 6), and CA (23.3%; *n* = 10) cohort (*p* < 0.001) (Table 1). Low-grade tumors were seldom observed in the Nigerian (17.9%; *n* = 5), Barbadian (0%; *n* = 0), and AA (10.0%; *n* = 2) cohorts compared to the CA (30.2%; *n* = 13) cohort (*p* < 0.0001; Table 1). Low-grade was defined as grade 1, medium-grade as grade 2, and high-grade as grade 3, respectively. Approximately 39.3% (*n* = 11) of Nigerian women presented with higher stage (T3–T4) tumors compared with 2.2% (*n* = 1) for Barbadian, 5.0% (*n* = 1) for AA, and 0% (*n* = 0) for CA women (*p* < 0.0001; Table 1). Finally, CA TNBC patients displayed a higher frequency of lymph node-negative tumors (60.5%; *n* = 26) compared with that observed in Nigerian (14.3%; *n* = 4), Barbadian (23.9%; *n* = 11), and AA (35.0%; *n* = 7) TNBC patients (*p* = 0.02; Table 1).

### Kaiso is highly expressed in TNBC tissues of WAA compared to Caucasian women

**Fig. 2:**
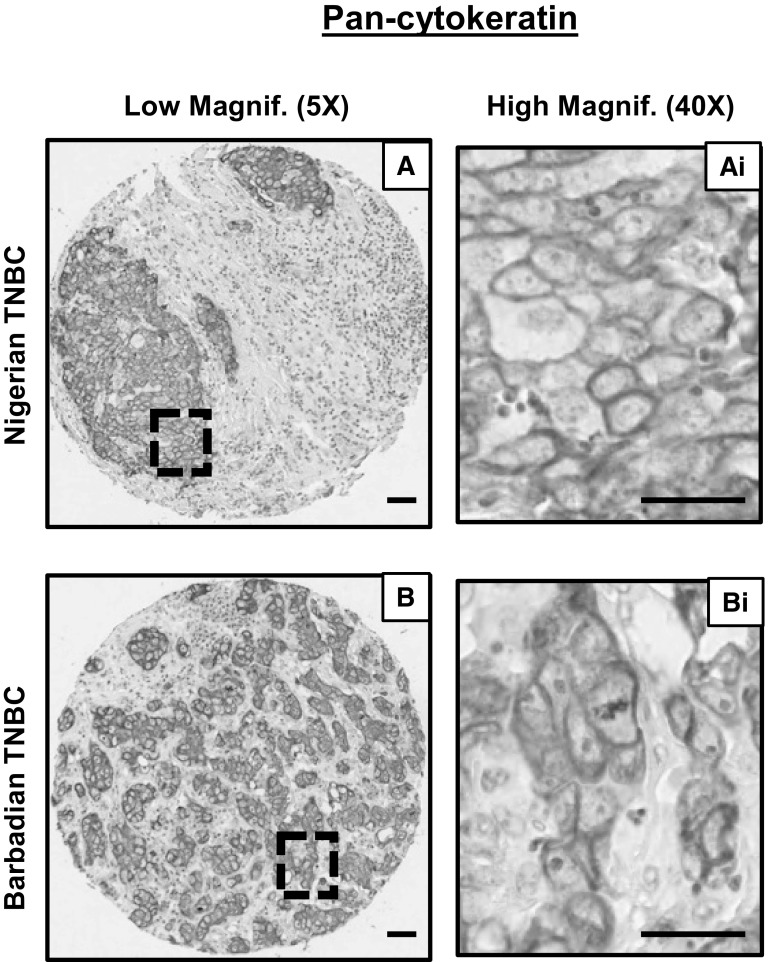
Cytokeratin immunostaining of Nigerian and Barbadian TNBC tissues verifies tissue integrity. IHC images at low (5×) and high magnification (40×) show intact tissue cores (**a**, **b**) and membrane localization (**a**i, **b**i) of cytokeratin, which portrays good integrity of the Nigerian and Barbadian tissues. *Scale bar* 50 μm

**Fig. 3:**
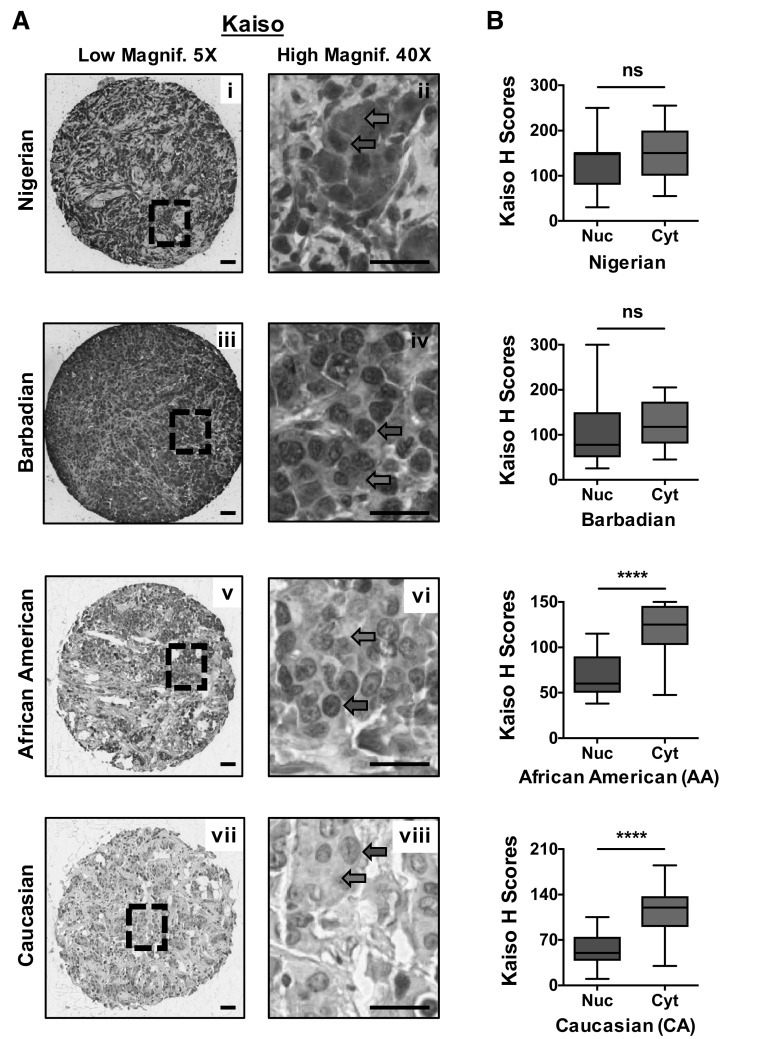
Kaiso subcellular localization and expression in Nigerian, Barbadian, AA, and CA TNBC tissues. (**a**i–viii) IHC images showing Kaiso localization to both the nucleus and cytoplasm of Nigerian, Barbadian, AA, and CA TNBC tissues. (**b**) Graphical representation of nuclear and cytoplasmic Kaiso expression in Nigerian (*n* = 19), Barbadian (*n* = 20), AA (*n* = 20), and CA (*n* = 39) TNBC tissues. Cytoplasmic Kaiso expression was significantly higher than nuclear Kaiso expression in the AA and CA TNBC cohorts but not in the Nigerian and Barbadian TNBC cohorts. Red arrows indicate nuclear Kaiso staining, while blue arrows indicate cytoplasmic Kaiso staining. *Scale bar* 50 μm. *ns* not significant, ^****^ *p* < 0.0001

Previously, we reported that Kaiso is highly expressed at the mRNA level in triple-negative tumors compared with hormone receptor-positive breast tumors in publicly available datasets downloaded from The Cancer Genome Atlas—TCGA website or the Gene Expression Omnibus—GEO website [35]. Thus, in this study, we utilized immunohistochemistry to specifically evaluate the expression and subcellular localization of Kaiso in TNBC tissues from Nigerian, Barbadian, AA, and CA patients. Tissue integrity of the Nigerian and Barbadian TNBC tissues was determined by immunostaining for pan-cytokeratin as described in the methods; Fig. 2a, b shows representative images of the tissue quality of the Nigerian and Barbadian TNBC tissues. As shown in Fig. 3a (representative images shown), Kaiso exhibited both nuclear and cytoplasmic localization in all TNBC tissues analyzed, with varying degrees of heterogeneity. Nuclear and cytoplasmic Kaiso staining intensity was scored as described in the methods, and Kaiso’s relative expression in each TNBC cohort analyzed. As seen in Fig. 3b, we observed significantly higher cytoplasmic than nuclear Kaiso expression in the AA and CA TNBC cohorts (*p* < 0.0001), but did not find significant differences between nuclear and cytoplasmic Kaiso expression in the Nigerian and Barbadian TNBC cohorts.

**Fig. 4:**
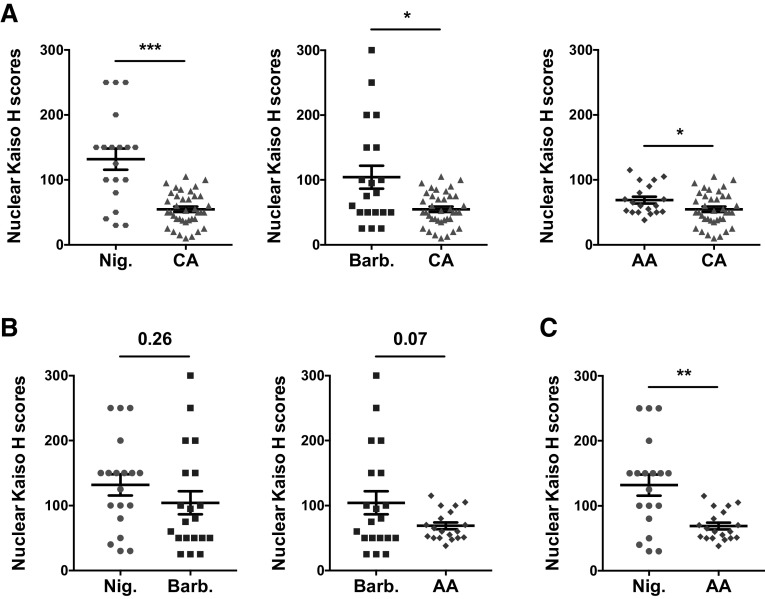
Comparative analysis of nuclear Kaiso expression in Nigerian, Barbadian, AA, and CA TNBC tissues. Higher levels of nuclear Kaiso expression were detected in TNBC tissues of Nigerian, Barbadian, and AA compared with their Caucasian counterparts (**a**). Although no significant difference in nuclear Kaiso expression was observed between Nigerian versus Barbadian tissues, or between Barbadian versus AA tissues (**b**), there was a significant difference in nuclear Kaiso expression between Nigerian and AA TNBC tissues (**c**). ^*^ *p* < 0.05, ^**^ *p* < 0.005, ^***^ *p* < 0.001

Since nuclear but not cytoplasmic Kaiso expression is known to be associated with TNBC aggressiveness, and decreased survival of AA BCa patients [19, 38], we next performed comparative analysis of nuclear Kaiso expression between the Nigerian, Barbadian, AA, and CA cohorts. Interestingly, we observed a significantly higher level of nuclear Kaiso expression in TNBC tissues of patients of African ancestry (Nigerian, Barbadian, and AA) compared to their Caucasian counterparts (Fig. 4a). However, there was no significant difference between nuclear Kaiso expression in TNBC tissues of Nigerian and Barbadian patients, who have ~99.8 and ~77.4% degree of African heritage, respectively [41, 42], or between TNBC tissues of Barbadian and AA patients, who have ~77.4 and ~72.5% degree of African heritage, respectively [42] (Fig. 4b). Remarkably however, there was significantly more nuclear Kaiso expression in TNBC tissues of Nigerian compared to AA patients (Fig. 4c), probably due to the higher degree of African heritage in Nigerian patients (~99.8%) compared to AA patients (~72.5%). Since TNBC is more prevalent in WAA compared to Caucasian women, these findings suggest a role for nuclear Kaiso expression levels in the racial disparity in TNBC prevalence.

### Correlation between nuclear Kaiso expression and clinico-pathological features of study participants

Breast tumors of WAA are often associated with a higher histological grade and positive lymph node involvement compared to breast tumors of Caucasian women [11, 14]. Since previous studies from our lab and others have correlated increased Kaiso expression with advanced grade and metastasis of TNBC [35, 38], and lymph node involvement is an established prognostic marker for the metastatic potential of breast tumors [43], we next assessed the association of Kaiso expression with high-grade and lymph node involvement in Nigerian, Barbadian, AA, and CA patients. High-grade tumors were defined as grade 3 for Nigerian and Barbadian patients and grade 2 for AA and CA patients due to no analyzed grade 3 tumors in the AA and CA TNBC cohort (the only observed grade 3 CA patient could not be scored as a result of tissue loss). Low-grade tumors were thus defined as grades 1 and 2 for Nigerian and Barbadian patients, and grade 1 for AA and CA patients. Lymph node metastasis was considered positive if one or more lymph nodes were noted to contain cancer cells (n1–n3), and negative if there were no observed cancer cells in the lymph nodes (n0). Due to the small sample size used in the analysis, no significant correlation was found between high nuclear Kaiso expression and high-grade or lymph node-positive triple-negative tumors in any of the patient cohorts analyzed (Suppl. Figure 1).

## Discussion

TNBC is most prevalent in WAA compared to Caucasian American/European females, but the reason for this disparity is currently unknown [11, 14, 16, 44]. Although poor socio-economic status has been linked to TNBC mortality in African and AA women, it does not fully explain the disproportionate prevalence and aggressiveness of TNBC in WAA compared to their Caucasian counterparts [17]. Thus, we and others have postulated that there may be an ancestral genetic predisposition to TNBC in WAA [17, 45].

Notably, a higher prevalence of TNBC has been reported in West-African women (Nigerians—65%, and Ghanaians—82.2%) compared with that reported in AA— ~33% [9, 11, 46], thus supporting the idea of a relationship between percentage of African ancestry and TNBC prevalence. Since West-African countries such as Ghana and Nigeria are the founding ancestors of most WAA worldwide [41, 42, 47–49], we posit that there is a higher probability of identifying a founder mutation, if one exists, in Nigerian and Ghanaian populations, and also in more homogeneous populations of the African Diaspora such as the Caribbean (e.g., Barbados).

Recent studies have linked high nuclear expression of the transcription factor Kaiso with increased TNBC aggressiveness [20, 38], and decreased survival of AA breast cancer patients compared with their Caucasian counterparts [19]. These reports suggest a link between increased nuclear Kaiso, TNBC aggressiveness/metastasis, and the racial disparity in prevalence/outcomes associated with breast cancer. Remarkably, our findings lend some credence to this hypothesis as we observed elevated expression of nuclear Kaiso in TNBC tissues from patients of African ancestry (Nigerians, Barbadians, and African Americans) compared to their Caucasian/European ancestry counterparts (CA) (see Fig. 4a). Thus, our previous findings in Kaiso-depleted mouse xenograft models [35, 51], where we demonstrated roles for Kaiso in TNBC cell growth, survival, and metastasis, may explain why high Kaiso-expressing triple-negative tumors in WAA are associated with a more aggressive phenotype and fatal outcomes than TNBC in Caucasian women.

Importantly, our findings highlight an interesting correlation between high nuclear Kaiso expression and percent African ancestry, which may be linked to the predisposition of young WAA to TNBC. However, this study is limited by the small sample size, the semi-quantitative method of analysis used, and lack of complete clinico-pathological information, which did not allow proper assessment of the correlation between Kaiso expression and the high tumor grade observed in African/Caribbean women compared to African American or Caucasian women. Additional studies using larger cohort sizes of West-African (Nigeria and others), Caribbean (Barbados and others), AA, and CA TNBC cases, coupled with quantitative methods of immunostain analysis such as the automated quantitative analysis (AQUA) system established by Rimm and colleagues [50], will undoubtedly provide more insight into the clinical relevance of nuclear Kaiso expression in the etiology of TNBC in WAA.

In conclusion, this is the first study to suggest a potential link between increased Kaiso expression and the predisposition of young WAA to TNBC. This observation, in addition to the previous identified roles for Kaiso in TNBC aggressiveness, metastasis, and poor overall survival in affected patients [35, 38, 51], raises two exciting possibilities: i) Kaiso expression could be utilized as a biomarker for the diagnosis and prognosis of TNBC in WAA and ii) Kaiso could be a molecular target for the development of treatment options against TNBC not only in WAA but also TNBC patients worldwide.

## Electronic supplementary material

Below is the link to the electronic supplementary material.

Supplementary material 1 (DOCX 223 kb)

Supplementary material 2 (DOCX 233 kb)
