# Supplementary material for: Kaiso is highly expressed in TNBC tissues of women of African ancestry compared to Caucasian women
Source: Cancer Causes Control. 2017 Sep 8;28(11):1295–304. doi: 10.1007/s10552-017-0955-2 (PMC5681979; doi:10.1007/s10552-017-0955-2)
Supplement: Supplementary file 1 — Supplementary material 1 (DOCX 223 kb) [file 10552_2017_955_MOESM1_ESM.docx]

**Suppl. Figure 1: High nuclear Kaiso expression is modestly associated with advanced grade and lymph node involvement of Nigerian, Barbadian and AA but not CA triple negative tumors.** (**a**) Graphical representation of nuclear Kaiso expression in **low**-**g**rade (low G) and **high**-**g**rade (high G) tumors of Nigerian (n total = 16), Barbadian (n total = 18), AA (n total = 12) and CA (n total = 31) TNBC patients. (**b**) Graphical representation of nuclear Kaiso expression in **l**ymph **n**ode **neg**ative (LN-neg) and **l**ymph **n**ode **pos**itive (LN-pos) tumors of Nigerian (n total = 11), Barbadian (n total = 11), AA (n total = 19) and CA (n total = 34) TNBC patients. A modest increase in nuclear Kaiso expression is observed in high-grade tumors of Barbadian and AA patients compared to their non-high-grade counterparts, and lymph node positive tumors of Nigerian and AA patients compared to their lymph node negative counterparts. No difference in nuclear Kaiso expression is observed in high versus low-grade tumors, and in lymph node positive versus negative tumors of CA patients. ns = **n**ot **s**ignificant

**Bassey-Archibong et al. Suppl. Figure 1**
