# Supplementary figures and images for: Kaiso is highly expressed in TNBC tissues of women of African ancestry compared to Caucasian women
Source: Cancer Causes Control. 2017 Sep 8;28(11):1295–304. doi: 10.1007/s10552-017-0955-2 (PMC5681979; doi:10.1007/s10552-017-0955-2)

**Averaged Pathologists’ scores used for Kaiso TNBC TMA analysis**


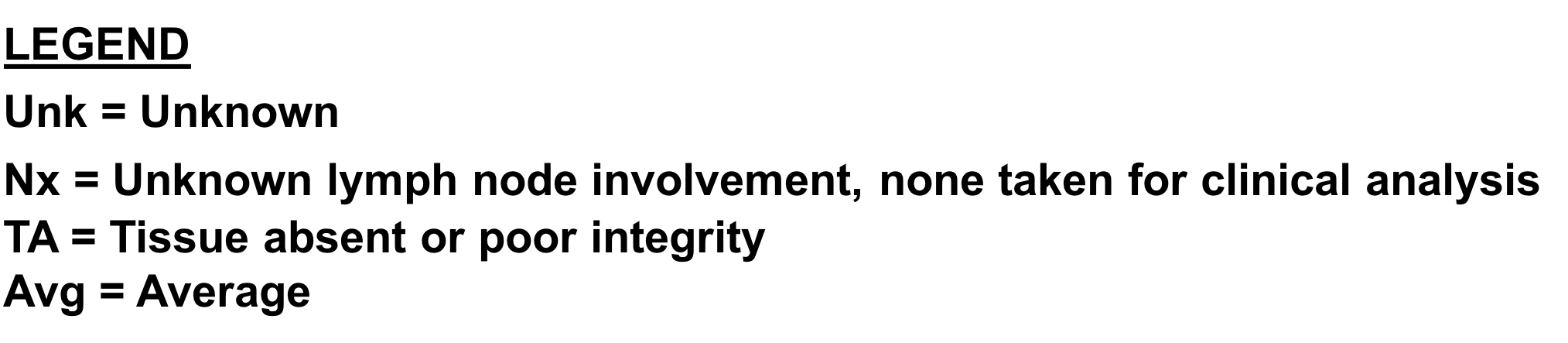

Supplement: Supplementary file 2 — Supplementary material 2 (DOCX 233 kb) [file 10552_2017_955_MOESM2_ESM.docx]
